# Further Information Respecting the Case of Mrs. Craib, Formerly Announced as Cured of a Cancer in Both Breasts by Dr. Nisbet. With Additional Cases

**Published:** 1801-02

**Authors:** Isaac Oliphant

**Affiliations:** Percy Street


### *Further Information respecting the Case of Mrs. Craib, formerly announced as cured of a Cancer in both Breasts by Dr. Nisbet. With additional Cases.* Communicated by Mr. Oliphant.

To the Editors of the Medical and Physical Journal.

Gentlemen,

In the laſt Number of your Journal, there appears a "Reply to Mr. Oliphant's Account of Mrs. Craib's Caſe of Cancer," wherein it is ſtated, that my Communication was "clearly meant to imply a reflection on the former relation;" whereas my motive, I ſolemnly declare, was what I thought due to myſelf and the public on this occaſion, viz. ſolely to give a faithful detail of the caſe of Mrs. Craib; which I did, and ſincerely conſole myſelf in ſo doing, for reaſons hereafter mentioned.

I cannot help obſerving, in order to leſſen the dependance on my authenticity, and to draw aſide the public's notice from my report of the caſe, ſo eſſentially different from his, that much ingenuity has been employed by Dr. Niſbet to ſerve his purpoſe; this, with thoſe who know me, will weigh nothing; and will weigh juſt as much with others when they are acquainted with the ſequel.

The gentlemen advertiſed in his firſt communication had very little to do with the detail of Mrs. Craib's caſe; therefore I conſidered particular notice was irrelevant in my report; only two preſcribed, the reſt merely looked at it; they did not all give an opinion: yet an effort has been made to degrade their profeſſional abilities before the public, under his ſpecious pretence, merely to give them "an opportunity of proving the authenticity of the ſtatement;" which, I believe, they did not give themſelves the trouble to do; whereas, in fact, it was to enrich his own triumph and influence in the public opinion. When there were collected together, by private invitation, ſeveral other gentlemen of the profeſſion, to dandle this abortive unique, ſurely the like gentle mode might have been extended to the other reſpectable ſurgeons, if a ſimple opportunity had been alone in contemplation: No, it could not here be ſo intended, though

"Inter ſe convenit urſis."

My intention being a detail of facts, not controverſy about imaginary points, however wiſhed for, in the relative deſcription of diſeaſed appearances, the moſt eſſential difference will be be ſeen: No reverted edges, no pouring out of quantities of blood; no rheumatic or erratic pains, except a few hours of pain from very hard labour; no violent pain in the breaſt, or large ulcer, ever happened: there were ſome alarming chronic inflammations in the miliary glands of the ſkin, and a red ſub-livid hue of the whole breaſt; the laſt was the conſequence of accident and empirical treatment.

I did not object to the frictions, in the firſt inſtance: Being reſponſible for a ſafe conduct of treatment, when my patient was liſtleſs and greatly reduced, with loſs of appetite, and a ſore mouth, in extremely hot weather, not favourable to the regaining of loſt ſtrength, by her ſolicitation, and my own opinion, I deſired that the ſevere diſcipline might be laid aſide. There was no ſhrinking of the breaſt perceptible, but what was in common to the reſt of the body, except as far as aroſe from an extenſion of the ulcerations, till after the pleuritic attack.

The reſolution of the upper and larger part of the diſeaſed breaſt into matter, was often ſaid to be at hand, and in an actual ſtate of ſuppuration, though it never happened; though felt, it was never ſeen.

Of the primary cauſe of abſorption of the breaſt, and the altered condition of the ulcerations, I have given an opinion in my ſtatement; wherein I neither took the merit of a cure to myſelf, nor allowed it to the Doctor: though I admitted the breaſt was abſorbed, and the ulcers healed, yet a conſtitutional diſeaſed irritation ſubſiſted from this abſorption.

If it is meant, by the "impropriety of the application," (pronounced, I am ſure, by no oracle in phyſic,) that my treatment of the patient in the latter week of October was improper, when I conſidered it uſeleſs to conſult him, I muſt ſay, (by the violent interpoſition of Dr. Niſbet, who, unſolicited, called in another phyſician to give weight, and carry the patient by a coup de main,) a decoction of Anguſtura bark, with vitriolic acid, was preſcribed by him; and though the practice was irregular, I made up this medicine, having alone a wiſh to benefit the patient, and to convince her that ſhe ſhould have all the aſſiſtance ſuggeſted for relief: I gave her, however, my opinion, on adminiſtering the firſt doſe. By this treatment a ſtop was put to the plentiful glary expectoration; a moſt teaſing cough came on; tightneſs and oppreſſion acroſs the cheſt, with ſhort breath; ſo that I had the greateſt difficulty to perſuade her to repeat the doſe, and only on the aſſurance that ſhe ſhould have a large delaxant portion of ipecacuanha, with opium, and ſome volatile alkali, to provide againſt ſerious conſequences, ſhould they enſue; a ſecond portion was given, but might have proved fatal, (according to the account of her friends friends who attended, and Mrs. Craib herfelf, the following morning,) if fhe had not taken the provided medicine. Though the Doctor fuppofed he had cured the cancer, he pufhed further here in the province of medicine.

Did I treat my cafes "with bark and carbonic acid?" Has Dr. Nifbet compiled fo much, and yet in a trifling ftatement is capable of making fuch a miftake? This error is of itfelf fufpicious. However, I fhould not be furprifed to find the greateft enemy to bark in detail, employ it in practice, and without reftriction: fuch to me feems to be the cafe with opium.

I muft take notice alfo, that the reflector on Mrs. Ofborn's cafe has paid lefs attention to the circumftances of it, than to his own reveries: the fkin was puckered before inflammation, fuppuration, or floughing; but after floughing, the referved fkin extended fmoothly over the formerly ulcerated furface, not healing as a fcrofulous fore, the appearance of which every tyro, nay, every old woman is acquainted with.

As to his making a comparifon between the influence of fudden inanition and ftarvation, in removing morbid accumulation by abforption, it is only an interlude.

It is true, Dr. Nifbet afked me, if I had any objection to his giving Mrs. Craib's cafe to fome periodical publication; and I faid, none. When he had written it, he waited on me in his way to the receiver of communications, and read it over. To the beft of my recollection nothing particularly paffed, except at our parting from my houfe, wherein I told him, his account, with regard to my opinion given to Mrs. Craib, when I firft faw her, was not true; and a proof of this he could have from herfelf. My opinion was, that I gave her hopes of a floughing of the whole difeafed breaft, not "by abforption," as was afterwards inferted; and alfo an improbability of any of it being reftored to health. Before he read me his account, as in the other reports of opinions, it was reprefented, in the moft unqualified pronunciation, to be incurable, and that he of himfelf had performed a perfect cure. Whether he would have made any alteration from what I faid, I knew not: indeed, it was of no confequence.

Mr. Coe, Mrs. Craib's friend, and Dr. Nisbet's operator, a worthy man, though in a humble ftation of life, called upon me the fame evening; and it was mentioned to him what his acquaintance Dr. Nifbet intended; the manner and precipitancy of which I difapproved of. He faid he would go to the Doctor, as it ftruck him in the fame way; and would talk to him about it. He called again, and told me the Doctor replied, "he would ftop its publication."

What

What right has any one to judge of another profeffional man's fentiments, who had not himfelf purfued "the candid line of conduct," and had broken off all communion by his empirical conduct, and reduced himfelf below the level of a regular practitioner?

With regard to joint names, was it likely I would make myfelf refponfible for any thing Dr. Nifbet might have chofen to infert? for, in fact, I had not even the reading of it; yet I was to bear its fate for the Doctor's fake. This is a fac fimile to his declaring, in a note to Dr. Bradley, that leave was given, by gentlemen who had feen the cafe, to atteft the cure by the ufe of their names: whereas, in truth, Mr. BLAIR, one of them, did afterwards publifh a gentle rebuke and refutation of fuch a tender on his part.*

As far as hitherto concerns our ftatement of Mrs. Craib's cafe to the public, the patient herfelf had read both; and was afked, in Mr. BLAIR's prefence, her opinion with regard to their juftnefs: Her anfwer was, "If fhe had not been told Dr. Nifbet's ftatement was that of her diforder, fhe could not have thought it; but that, as far as fhe could judge, or knew, Mr. Oliphant's account was juft."

My ftatement in the London Medical Review and Magazine for December, fo far from advancing that I had cured the patient, according to Dr. Nifbet's attack, will be found to contain thefe words: "However, fubfifting ftill is a *scirrhus* in the right breaft, which feems to enlarge with her repletion; and there is not the leaft doubt of morbid irritation exifting in her habit, moft likely the offspring of her abforbed difeafed breafts, or that difpofition which produces fcirrhus."

I am forry my opinion has been realized. On the 1ft of this month fhe fent for me, to fhew me a few eruptions that had come out the preceding fortnight; and with confiderable relief to her infide, fhe told me. When I faw her, I found there were innumerable affections of the miliary glands of the fkin of both breafts, particularly under the abforbed one, extending a great way on the fide under the arm. The laft healed fore, where the abforbed one was, was re-opened; on feveral other parts of this furface confiderable fcirrhous tubercles have arifen. The right breaft, from being left with a fmall pendulous fcirrhus, fuftained by the flaccid integuments; the fkin has now contracted, which draws up the increafed fcirrhus; and the whole threatens to become one mafs of difeafe, of much more ferious confideration to Mrs. Craib than any

* Medical and Phyfical Journal, No. XXII. p. 569.

any former ſtate of her malady that I have witneſſed: as alſo her preſent weakneſs, indifferent appetite, bad digeſtion, a diſpoſition to anaſarca, and her pulſe upwards of 112, have diſabled the conſtitution from ſuſtaining ſuch a grievous load for any great length of time.

Leſt this farther account of my patient's caſe ſhould be called in queſtion, from ſiniſter motives, as had already happened, I requeſted Mr. Blair to ſee the ſtate of Mrs. Craib's health on the 8th inſtant; which he politely and obligingly did, and favoured me with his report of what he ſaw, as follows:

A Letter from Mr. Blair to Mr. Oliphant.

"Sir,

"As you have done me the honour to give me a ſight of Mrs. Craib's caſe, I cannot reaſonably decline writing (as you deſire) a ſhort ſtatement of what I then witneſſed.

"When I formerly ſaw the patient with Dr. Niſbet, appearances ſeemed very much in her favour, and there was even room to hope for a complete cure; but, yeſterday, I obſerved the glandular enlargement and induration of the right breaſt had increaſed, the ſkin and integuments of the left ſide were irregular, tuberculated, and adhering to the pectoral muſcle; beſides which there were various hardened ſubcutaneous glands about the left breaſt, extending towards the armpit.

"I here beg leave to conclude my evidence. If it will ſerve to throw light on the truth, you are welcome to make uſe of this letter. I remain,

"Sir,

"Your obedient humble ſervant,

"William Blair."

*Great Ruſsel Street, Bloomsbury,*

*January* 9, 1801.

The following two caſes, which I am afraid will be followed by ſeveral others, will further afford a reaſonable juſtification for my giving the fair ſtatement of Mrs. Craib's caſe.

In February laſt Charlotte Logan, at Mr. Campbell's, Newcaſtle Street, Strand, aged 46, had her menſtrua ſuddenly ſuppreſſed apparently by a violent emotion of the mind; and in the ſummer perceived a ſmall ſcirrhus in her left breaſt, which became painful in Auguſt following.

About September 25th ſhe was attacked with fever, ſaid by the gentleman who attended her, to be independent of the condition of the breaſt; but on the incumbent ſkin of it, and to a great extent on the left ſide, a diffuſed redneſs appeared, attended with much pain, which was removed by repeated bleedings with leeches, and left the ſcirrhus in the ſame condition as before.

From

From the alarming ftate of the breaft, fhe was advifed to go into St. Bartholomew's Hofpital, wherein fhe remained nearly a fortnight. After a confultation of the furgeons, excifion of the difeafed part was concluded on: Hearing, however, of Dr. Nifbet's cure of Mrs. Craib in the beginning of October, her friends removed her fuddenly to be under his care, who pronounced "he would cure her, at fartheft, in fix weeks."

On the feventh week, it was faid that the breaft would fuppurate and burft in eight days. But a few more days were paffed; and fuppuration did not follow.

After being nine weeks under his frictions, fhe was taken with a violent pain in her head, when fhe was defired to keep her bed. For a fortnight fhe was confined: The Doctor only faw her once, and never after returned, for reafons beft known to himfelf, having attended her eleven weeks. There was great increafe of the pain and difeafe in the breaft, and an oozing from around the nipple, forming troublefome incruftations.

A week after this fhe applied to me, and I found the whole left breaft fcirrhous, feveral cuticular glands inflamed, which appeared firft under the Doctor's care, and an incruftation round the nipple; the whole attended with moft excruciating pain, and vaft agitation of mind. She now wanted me to pronounce whether it could poffibly be cured: Not being bold enough to fatisfy her wifhes, fhe was put upon a plan that was confidered as too expenfive in the purchafe of malt.— Though the application foothed the breaft, yet I did not fee her after, till I met her on the 1ft inftant at Mrs. Craib's for the fecond time, and found fhe had an ointment fpread on paper: I therefore conclude fhe is now under the direction of fome old woman. During this interval feveral lymphatic glands behind the clavicle, and up the fide of her neck, have become enlarged.

There is alfo the cafe of Mrs. ———, who put herfelf under the care of Dr. Nifbet, in confequence of the firft publication of Mrs. Craib's cafe; but the refult of that can be "beft related by the undertaker."

I have now, Gentlemen, laid before you and the public all the facts deferving their notice refpecting Mrs. Craib's cafe. It was very much hoped for, that perfonality might have been avoided: However, unfortunately a fpirit has fhewn itfelf, living and thriving on controverfy, that has dragged me into meafures I highly diflike, and I muft, in future, leave to itfelf, a fpirit which wifhes to fubftitute fophiftry for plain facts, difhonourable infinuation for claim of rectitude, and fcholaftic reafoning for practical opinion. I am, &c.

